# The Lymphatic Endothelial Cell Secretome Inhibits Osteoblast Differentiation and Bone Formation

**DOI:** 10.3390/cells12202482

**Published:** 2023-10-18

**Authors:** Ernesto Solorzano, Andrew L. Alejo, Hope C. Ball, Gabrielle T. Robinson, Andrea L. Solorzano, Rama Safadi, Jacob Douglas, Michael Kelly, Fayez F. Safadi

**Affiliations:** 1 Department of Anatomy and Neurobiology, Northeast Ohio Medical University (NEOMED), Rootstown, OH 44272, USA; esolorzanozepeda@neomed.edu (E.S.); aalejo@neomed.edu (A.L.A.); hball@neomed.edu (H.C.B.); grobinson@neomed.edu (G.T.R.); asolo048@fui.edu (A.L.S.); 2 Musculoskeletal Research Group, NEOMED, Rootstown, OH 44272, USA; jdouglas2@neomed.edu; 3 Basic and Translational Biomedicine (BTB) Graduate Program, College of Graduate Studies, NEOMED, Rootstown, OH 44272, USA; kellym19@ccf.org; 4 College of Arts and Sciences, Kent State University, Kent, OH 44243, USA; rsafadi@kent.edu; 5 Department of Pediatric Hematology Oncology and Blood, Cleveland Clinic, Cleveland, OH 44195, USA; 6 Rebecca D. Considine Research Institute, Akron Children’s Hospital, Akron, OH 44308, USA

**Keywords:** complex lymphatic anomaly, osteoblast, lymphatic endothelial cell, conditioned medium, bone, Gorham–Stout disease, osteopathy, bone formation

## Abstract

Complex lymphatic anomalies (CLAs) are a set of rare diseases with unique osteopathic profiles. Recent efforts have identified how lymphatic-specific somatic activating mutations can induce abnormal lymphatic formations that are capable of invading bone and inducing bone resorption. The abnormal bone resorption in CLA patients has been linked to overactive osteoclasts in areas with lymphatic invasions. Despite these findings, the mechanism associated with progressive bone loss in CLAs remains to be elucidated. In order to determine the role of osteoblasts in CLAs, we sought to assess osteoblast differentiation and bone formation when exposed to the lymphatic endothelial cell secretome. When treated with lymphatic endothelial cell conditioned medium (L-CM), osteoblasts exhibited a significant decrease in proliferation, differentiation, and function. Additionally, L-CM treatment also inhibited bone formation through a neonatal calvaria explant culture. These findings are the first to reveal how osteoblasts may be actively suppressed during bone lymphatic invasion in CLAs.

## 1. Introduction

Complex lymphatic anomalies (CLAs) are categorized by idiopathic boney lesions because of abnormal lymphatic invasion [1]. CLAs include Gorham–Stout disease (GSD), generalized lymphatic anomaly (GLA), kaposiform lymphangiomatosis (KLA), and central conducting lymphatic anomaly (CCLA). In CLAs, it is hypothesized that lymphatic invasion disrupts bone homeostasis towards bone resorption. In each CLA subtype, there is a unique osteopathic profile with a constant hindrance to bone regeneration in the affected regions. To best describe GSD-related osteopathies, eight unique key criteria have been proposed [2]. One of these criteria specifically notes the lack of bone regeneration, which allows for the massive bone destruction and resorption shown in boney phenotypes [3,4,5]. Each of these diseases shows varied levels of bone loss, such as trabecular osteolysis and channel-like osseous lesions, due to the presence of these abnormal lymphatics [6,7,8,9].

The lymphatic system is responsible for maintaining fluid homeostasis, housing the immune system, and lipid transport [10,11]. Under physiological conditions, initial lymphatics collect interstitial fluid into collecting vessels, which pump the lymph back into circulation [12]. Recent findings identified the presence of lymphatic vessels within bone under normal physiological conditions [13]. These findings suggest that dysregulation of these lymphatic vessels may play a critical role in the lymphatic malformations found in CLAs.

Bone homeostasis is maintained through a balance between bone resorption and bone formation, which are primarily overseen by osteoblasts (OBs) and osteoclasts (OCs), respectively. OBs originate from mesenchymal stem cells (MSCs), which have the ability to differentiate into various cell types, including osteocytes, osteoblasts, chondrocytes, and adipocytes [14]. OBs’ main function is to deposit minerals and mature the boney matrix. Runt-related transcription factor 2 (Runx2) and alkaline phosphatase (ALP) are two markers expressed by differentiating OBs as they build new bone [15,16]. OCs are multinucleated cells that originate from hematopoietic stem cells. Their main responsibility is bone resorption and remodeling. To achieve this, OCs form a sealing zone on bone and then secrete acidic enzymes that allow for bone matrix degradation [17,18].

Increased OC function and activity has been reported in various CLAs [19,20,21,22]. Lymphatic endothelial cells (LECs) invading bone have been hypothesized to originate from regional lymphatics proximal to the bone [23]. Mice overexpressing vascular endothelial growth factor-C (a lymphatic vessel inducer, VEGF-C) in osterix-expressing cells (OB lineage cells and chondrocytes) are believed to recruit lymphatic vessels through the cortex toward the intramedullary cavity, a process that requires OC function [24,25]. These studies support the hypothesis that the abnormal presence of lymphatic vessels within the bone leads to anomalous bone loss. Conversely, the effect of lymphatic invasion on OB function and activity has not been systematically studied. Since the relationship between OBs and OCs is well understood, it is advantageous to scrutinize OB function and activity when lymphatic vessels invade bone to further inspect potential mechanisms between abnormal lymphatics and bone homeostasis.

Intratibial injection of non-pathogenic LECs in mice led to massive bone loss within two weeks of injection [26]. This phenomenon was tightly linked to the overactivation of nascent OCs, which could be attributed to macrophage-colony-stimulating factor (OC growth factor) secretion from LECs [26]. GSD patients (known to exhibit massive bone loss) have been reported to have elevated serum levels of interleukin-6, CTX (a marker of bone collagen degradation), sclerostin, and VEGF-C, which are all markers of active bone resorption [27,28,29]. Together, these studies highlight how both lymphatic-to-bone contact and overproduction of bone resorption modulators may play key roles in CLA-related osteopathies.

**Figure 1:**
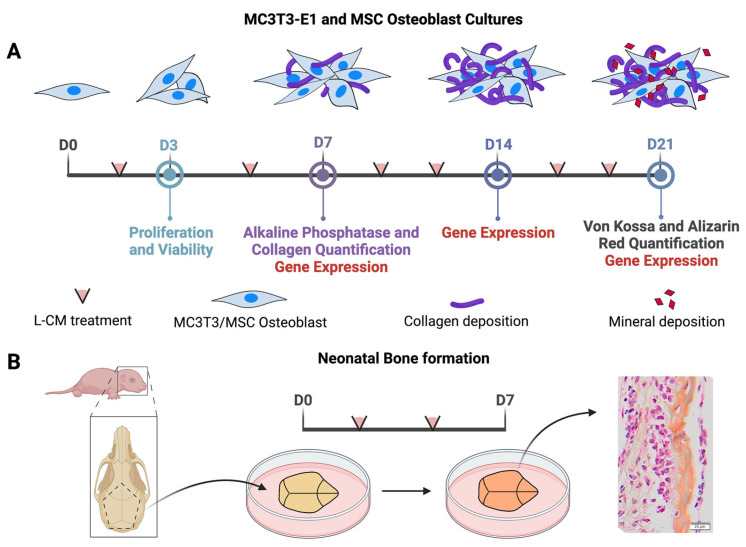
Schematic diagram of the experimental design. MC3T3-E1s or bone-marrow-derived MSCs were treated with L-CM and assessed in various analyses after 3, 7, 14, and 21 days of culture (**A**). Bone formation was assessed through an explant culture of neonatal calvaria when treated with L-CM and was quantified through histological analysis (**B**).

In this study, we hypothesize that factors secreted from lymphatic endothelial cells negatively regulate osteoblast differentiation and function, and inhibit bone formation in organ culture (Figure 1). OBs (MC3T3-E1s and bone-marrow-derived MSCs) were assessed for proliferation, viability, differentiation, function, and gene expression when cultured with varying concentrations of lymphatic endothelial cell conditioned medium (L-CM). Our results support the concept that factors secreted from LECs inhibit osteoblast differentiation and bone formation, as seen in CLAs.

## 2. Materials and Methods

### 2.1. Mice

Wild-type C57BL/6J male mice were purchased from Jackson Laboratories. The mouse colony was housed and maintained at Northeast Ohio Medical University (NEOMED) in a facility accredited by the Association for Assessment and Accreditation of Laboratory Animal Care International (AAALAC). Mouse housing was maintained at 21 °C with a 12 h light–dark cycle. The studies presented here were approved by the Institutional Animal Care and Use Committee (IACUC) at NEOMED.

### 2.2. Lymphatic Conditioned Medium (L-CM) Preparation

C57BL/6J mouse primary lymphatic endothelial cells were purchased from CellBiologics (Chicago, IL, USA) and passaged at least twice before being used for experiments. For the collection of lymphatic endothelial cell conditioned medium (L-CM), cells were grown to 70% confluence in T75 flasks with 10% fetal bovine serum (FBS) in α MEM medium (Gibco, Billings, MT, USA). Following this, cells were washed with PBS and treated with 15 mL of serum-free medium over 24 h. Serum-free conditioned medium was collected, centrifuged at 1000× *g* 5 min, decanted, and supplemented with fresh FBS (10 or 2%) depending on the experiment.

### 2.3. Proliferation and Viability

The calvaria-like OB MC3T3-E1 cell line (ATCC, Manassas, VA, USA) and MSCs derived from the bone marrow of femurs and tibias of C57BL/6J male mice at ages between 6 and 8 weeks were used for this study. Cells were cultured in 96-well plates and seeded at 5000 cells per well with 2% FBS while being treated with L-CM for 72 h. Proliferation was based on BrdU incorporation and was quantified using the CyQUANT^®^ NF Cell Proliferation Assay Kit (Invitrogen, Carlsbad, CA, USA) as per the manufacturer’s instructions. Cell proliferation was assessed using a fluorescent plate reader (excitation: 485 nm, emission: 530 nm, Spectrum SoftMax Pro version 6.5.1) [30]. For a viability assay, we used the MTT method. Cells were plated with methods similar to those stated above and were assessed using the CellTiter 96^®^ Aqueous One Solution Assay protocol (Promega, Madison, WI, USA). Plates were incubated with CellTiter 96^®^ and read at 490 nm using a 96-well plate reader (Spectrum SoftMax Pro) [31].

### 2.4. Isolation of Primary Osteoblasts and Differentiation

Murine bone marrow cells were flushed and collected from femurs and tibias of C57BL/6J male mice at ages between 6 and 8 weeks. Adherent cells were cultured in a 10 cm tissue culture dish. When the cells reached confluency, they were trypsinized, counted, and re-plated at a density of 5 × 10^5^ cells and 1 × 10^5^ cells in 6-well and 24-well plates, respectively. Cells were cultured in α MEM supplemented with 10% FBS, 50 μg/mL ascorbic acid, 10 mM β-glycerophosphate, and 10 nM dexamethasone (differentiation factors). Cell culture was terminated on days 7, 14, and 21 for RNA isolation. Staining for alkaline phosphatase and collagen was completed on day 7, while staining for minerals was conducted on day 21 in culture as previously described [32].

### 2.5. Alkaline Phosphatase (ALP) and Collagen Staining and Quantification

Early osteoblast differentiation was determined by ALP staining on day 7 as previously described [30,33,34]. ALP staining was prepared by mixing 10 mL of water, 5 mg of Fast Blue RR Salt, and 400 μL of AS-MX phosphate solution (Sigma, St. Louis, MO, USA). OB cultures were fixed with 10% formalin for 10 min, washed 3× with PBS, and exposed to ALP staining solution for 30 min while covered, followed by a PBS wash. Collagen staining was performed using a Sirius Red/Fast Green Collagen Staining Kit (Amsbio, Oxfordshire, UK) per the manufacturer’s instructions. OB cultures were fixed with Khale’s fixative, washed, and stained with dye solution for 30 min. Following imaging, the dye was lifted and read at OD values of 540 and 605 nm. Collagenous and non-collagenous content was quantified per the manufacturer’s instructions.

### 2.6. Von Kossa and Alizarin Red Staining and Quantification

Osteoblast matrix mineralization in cultures was determined by Von Kossa staining on day 21 as previously described [32]. OB cultures were stained with 2.5% silver nitrate solution under UV light for 30 min and then washed with water. Hydroxyapatite crystals of mineralized nodules were stained black and fixed with 5% sodium thiosulfate. Calcium-containing matrixes/cells were determined by Alizarin Red staining on day 21 as previously described [32]. OB cultures were fixed with 4% paraformaldehyde for 10 min, washed with PBS three times, and then incubated with Alizarin Red solution for 2–5 min. The dye was removed, and cells were rinsed with PBS five times and air dried. Quantification of Alizarin Red staining was performed by adding 200 μL of 10% acetic acid, followed by incubation while shaking. Cells were transferred to a 1.5 mL microcentrifuge tube, which was vigorously vortexed and heated to 85 °C for 10 min. The mixture was then placed on ice, followed by centrifugation at 20,000× *g* for 15 min. Then, 10% ammonium hydroxide was used to neutralize the pH, and 50 μL of Alizarin Red standard plus the sample was plated and read at 405 nm (OLYMPUS BX61VS).

### 2.7. RNA Isolation and RT-qPCR Analysis

RNA isolation from differentiated MC3T3-E1 cultures was performed using Qiazol reagent (miRNeasy Mini Kit, Qiagen, Hilden, Germany) on days 7, 14, and 21 as previously described [35]. RNA quantities and qualities were measured using a NanoDrop 2000 spectrophotometer (Thermo Fisher Scientific, Waltham, MA, USA). Complementary DNA was generated using a high-capacity cDNA reverse transcription kit (Applied Biosystems, Waltham, MA, USA). qPCR was performed with an ABI 7500 fast real-time PCR system (Life Technologies, Carlsbad, CA, USA) in triplicate (20 μL volume) per reaction for the following genes from each respective cell culture. MC3T3-E1 mRNA expression included ALP, collagen 1, Runx2, GPNMB (Osteoactivin), and RANK-L, and 18 s was used as the control gene. Each reaction contained 10 ng of cDNA, 100 nmol/L primers, and 10 μL of 2× SYBR Green PCR master mix (Life Technologies, Carlsbad, CA, USA). The primers used in these measurements are reported in Table 1.

### 2.8. Organ Culture, Staining, and Quantification

Neonatal C57BL/6J mice were sacrificed, and their calvariae were harvested between 3 and 4 days as described previously [36]. Under sterile conditions, mice calvariae were rinsed in a Petri dish with BGJ medium (Gibco, Billings, MT, USA) supplemented with 0.1% bovine serum albumin and 100 units/mL of penicillin and streptomycin (Sigma-Aldrich, St. Louis, MO, USA); then, by carefully using curved forceps and micro-scissors, they were dissected to reveal the various sutures of the skull. Cuts were made along the sagittal suture that passed through the coronal suture and ended at the anterior fontanelle. Then, straight cuts along the lambdoid sutures and 45-degree-angle cuts connecting both sides allowed for the removal of a pentagon-shaped calvaria. Each calvaria was cultured on a stainless-steel wire mesh grid, which was placed into the tissue culture wells with 1 mL of BGJ medium.

The calvariae were then processed (LEICA ASP300S, Leica Biosystems, Heidelberger, Germany) and embedded in paraffin (LEICA HistoCore Arcadia 4, Leica Biosystems, Heidelberger, Germany). Each block of paraffin was then sectioned (LEICA RMR235, Leica Biosystems, Heidelberger, Germany), trimmed to 7 μm sections of the sagittal suture, and placed on glass slides (Thermo Scientific, Waltham, MA, USA). The slides were stained using Alcian Blue Hematoxylin/Orange G stains to show bone cells (bright blue pericellular ring), cartilage (blue/purple), and bone (orange/red). The slides were hydrated with distilled water, placed in acid–alcohol for 20 s, and then soaked in the Alcian Blue Hematoxylin (Acros Organics, Waltham, MA, USA) for 10 min. The calvariae were washed gently with distilled water, dipped in acid–alcohol, and washed again with distilled water three times before being placed in 0.5% ammonium water (Fischer, Waldachtal, Germany) and being washed with distilled water again. The slides were then dipped in 95% ethanol (EtOH) for one minute and, finally, in the Eosin/Orange G solution (Sigma, St. Louis, MO, USA) for one minute before being dehydrated with three changes of 95% EtOH and two changes of 100% EtOH; then, they were mounted and coverslipped.

The slides were imaged (OLYMPUS VS-ASW-S6, Olympus Life Science, Waltham, MA, USA), and the coronal suture was identified first; then, under 20× magnification, two to three fields away from the coronal suture was determined to be the ideal area of bone to be analyzed as described [37]. Bone areas were calculated by tracing the borders of preexisting bone and new bone with the ImageJ software (Version 1.53).

### 2.9. Statistical Analysis

Data were analyzed using the GraphPad Prism 10.0.2 software (GraphPad, La Jolla, CA, USA). All individual experiments were repeated at a minimum of N = 3 per experiment with at least 3–6 replicates per experiment. One-way ANOVA followed by Dunnett’s post hoc test was performed when comparing multiple groups. An unpaired *t*-test was performed for comparisons of two groups. Differences were considered statistically significant when the *p*-value was less than 0.05. Group means or means ± standard error of the mean (±SEM) were graphed.

## 3. Results

### 3.1. L-CM Treatment Decreased MC3T3-E1 Cell Proliferation

**Figure 2:**
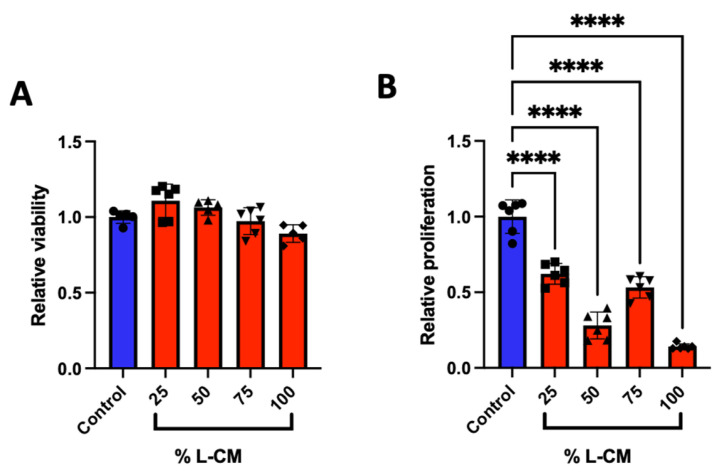
Lymphatic endothelial cell conditioned medium (L-CM) decreased MC3T3-E1 cell proliferation. MC3T3-E1 cells were cultured in a 96-well plate and assessed for cell viability (**A**) and cell proliferation (**B**) when treated with various doses of L-CM. N = 6. Data presented as mean ± SEM. **** *p* < 0.0001 compared to the untreated control.

GSD patients present an abnormal bone phenotype associated with decreased bone formation [2,3,4,5]. We investigated the role that the lymphatic secretome has on osteoblast viability and proliferation by treating an osteoblast-like cell line (MC3T3-E1) with various doses of lymphatic endothelial cell conditioned medium (L-CM). Using the MTT-based cell viability and DNA-binding proliferation assays, we treated MC3T3-E1 cells with 25, 50, 75, and 100% L-CM (Figure 2). While the effect on cell viability was not significant (Figure 2A), we found that L-CM negatively regulated MC3T3-E1 proliferation in a dose-dependent manner (Figure 2B). Given the inhibition of cell proliferation, we expect L-CM treatment to have an overall decrease in OB growth.

### 3.2. L-CM Treatment Inhibited Early Differentiation and Collagen Production in MC3T3-E1

**Figure 3:**
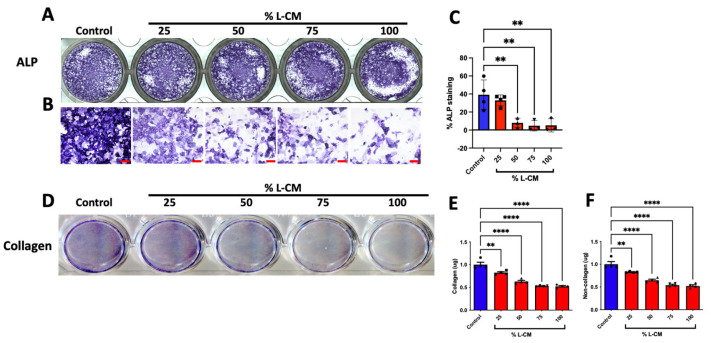
L-CM decreased early cell differentiation and function in MC3T3-E1 cells. MC3T3-E1 cells were cultured with differentiation factors and treated with 25, 50, 75, or 100% L-CM. After 1 week of culture, osteoblasts were stained for alkaline phosphatase (ALP) (**A**,**B**). ALP staining was then quantified and assessed for each treatment (**C**). Collagen production was assessed through staining (**D**) and then quantified for the content of collagenous (**E**) and non-collagenous (**F**) proteins. N = 3–4. Data are presented as the mean ± SEM. ** *p* < 0.01; **** *p* < 0.0001 compared to the untreated control. Scale bar: 100 μm.

To determine whether the effect of L-CM on the proliferation of MC3T3-E1 cells (Figure 2) was also reflected in their differentiation, we cultured MC3T3-E1 cells to confluence, followed by differentiation, and treated them with L-CM over the course of 7 days as previously described [32,33]. After 7 days of culture, MC3T3-E1 cells were terminated and stained for alkaline phosphatase (ALP) (Figure 3A,B), an early marker for osteoblast differentiation [38], which was followed by a quantification of the ALP-positive area fraction (Figure 3C). These data revealed an incremental decrease in ALP staining in response to increasing doses of L-CM.

Furthermore, we stained the collagen within the extracellular matrix and determined the quantities of collagenous and non-collagenous proteins (Figure 3D–F). As suspected from our ALP analysis, the extracellular matrix content progressively declined with increased concentrations of L-CM in both collagenous and non-collagenous proteins (Figure 3E,F). These results support the overall decrease in osteoblasts’ ability to synthetize their early extracellular matrix when exposed to L-CM, thus preventing proper early osteoblast function.

### 3.3. L-CM Treatment Inhibited MC3T3-E1 Matrix Mineral Deposition

**Figure 4:**
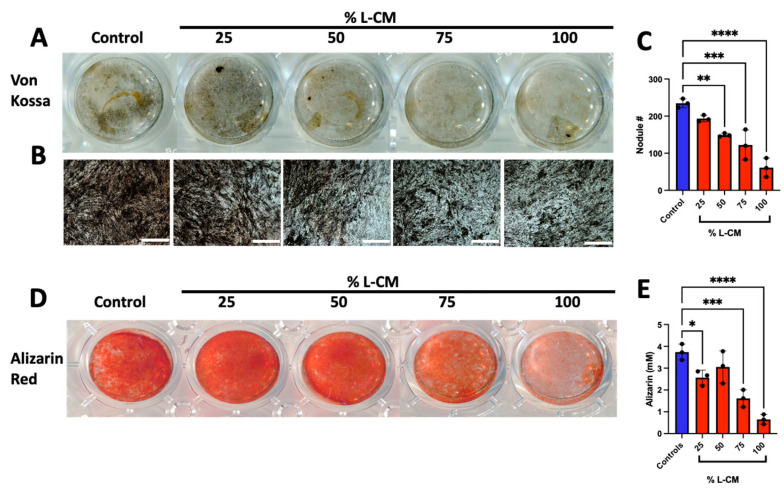
L-CM decreased mature MC3T3-E1 matrix mineral deposition. MC3T3-E1 cells were cultured with differentiation factors and treated with 25, 50, 75, or 100% L-CM. After 3 weeks in culture, osteoblasts were assessed for matrix mineralization and calcium deposition. Von Kossa staining was used to highlight deposits of calcium phosphate crystals (**A**,**B**), followed by the quantification of mineralized nodules (**C**). Calcium deposition was also determined using Alizarin Red staining (**D**) and quantification (**E**). N = 3. Data are presented as the mean ± SEM. * *p* < 0.05, ** *p* < 0.01, *** *p* < 0.001, and **** *p* < 0.0001 compared to the untreated controls. Scale bar: 1000 μm.

We next examined the effects of L-CM treatment on late osteoblast differentiation and function. MC3T3-E1 cells were differentiated in the presence of osteoblastic differentiation factors with continuous treatment of L-CM for a three-week period with every medium change. At day 21, differentiated MC3T3-E1 cells were assessed for mineral deposition, which is the hallmark function of mature osteoblasts, as described in [39,40,41] (Figure 4). Mineral deposits were stained using the Von Kossa staining protocol described in [41] (Figure 4A,B), followed by the quantification of mineralized nodules (a late osteoblast function marker) (Figure 4C).

Of the minerals that are secreted, calcium is the main component needed for bone mass maintenance [42]. Using the Alizarin Red assay, we stained and quantified calcium deposition in late-differentiated MC3T3-E1 cells when treated with varying doses of L-CM (Figure 4D,E). Our results present a significant decrease in calcium deposition with most L-CM doses. Using these approaches, we determined that L-CM not only decreased early-stage osteoblast differentiation, but also inhibited mineral deposition in mature osteoblasts in comparison with the untreated controls.

### 3.4. L-CM Treatment Decreased the Gene Expression of Osteoblast-Related Markers

**Figure 5:**
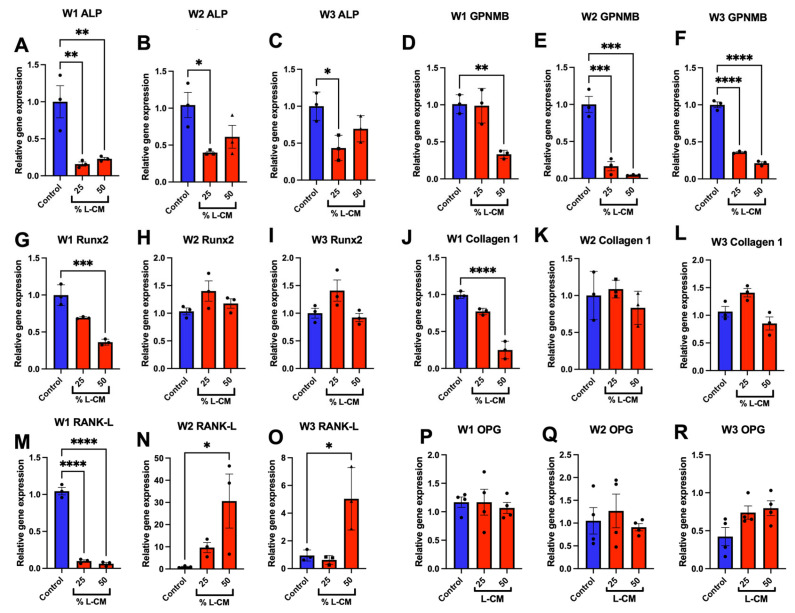
L-CM decreased the gene expression of osteoblast markers. MC3T3-E1 cells were differentiated with differentiation factors and treated with either 25 or 50% L-CM. The mRNA expression of osteoblast-related markers was assessed after one (W1), two (W2), and three (W3) weeks of culture (**A**–**R**). N = 3. Data are presented as the mean ± SEM. * *p* < 0.05, ** *p* < 0.01, *** *p* < 0.001, and **** *p* < 0.0001 compared to the untreated controls.

Following the differentiation of MC3T3-E1 cells treated with L-CM, RNA isolated after 7, 14, and 21 days of culture was reverse-transcribed to assess the mRNA expression of osteoblast-related markers. Here, we show that ALP mRNA expression was significantly decreased throughout all three weeks of differentiation (Figure 5A–C); this directly supports the results observed in Figure 3. The expression of Runx2 and collagen 1, which are markers of early osteoblast differentiation and function [43], was decreased by L-CM in the first week of differentiation (Figure 5G,J). In addition, we also found the decreased mRNA expression of GPNMB (osteoactivin), which has been found to be a positive regulator of osteoblast differentiation [30,32,33,44,45] (Figure 5D–F). Lastly, we assessed the mRNA expression of receptor activator of nuclear factor Kappa beta ligand (RANK-L), which is an osteokine secreted by osteoblasts that is necessary for osteoclast differentiation [46,47]. At week one, we found that L-CM blunted RANK-L mRNA expression (Figure 5M) [48]. Interestingly, this effect was significantly reversed beyond the control at two and three weeks in culture (Figure 5N,O). Lastly, the expression of osteoprotegerin (OPG), an inhibitor of RANK-L, showed no difference in comparison to the control. Combined with the decreased expression of osteoblast-related differentiation factors and increased expression of RANK-L, our data suggest that bone homeostasis might shift towards bone resorption with little to no new bone formation, as observed in GSD patients [2,5,49].

Together, these results have highlighted how factors secreted from murine lymphatic endothelial cells lead to decreased osteoblast differentiation, function, and gene expression. These results are in contrast with those of previously published work that described how L-CM did not have a significant impact on osteoblast differentiation in association with ALP staining and fraction quantification [26]. This group used differentiated bone-marrow-derived mesenchymal stem cells (MSCs) and did not find a significant difference after two days of differentiation. To further support our results, we assessed the effects of L-CM on osteoblasts derived from murine bone-marrow-derived MSCs.

### 3.5. L-CM Treatment Inhibited Bone-Marrow-Derived MSC Proliferation

**Figure 6:**
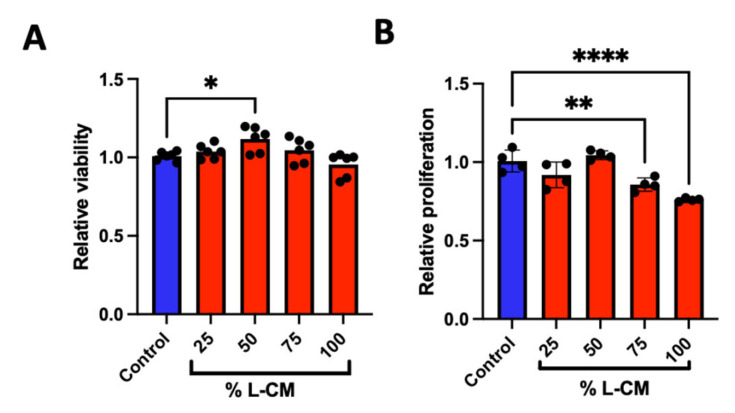
L-CM decreased mesenchymal stem cell (MSC) proliferation. Bone-marrow-derived MSCs were cultured and assessed for cell viability (**A**) and cell proliferation (**B**) after being treated with various doses of L-CM. N = 4–6. Data are presented as the mean ± SEM. * *p* < 0.05, ** *p* < 0.01, and **** *p* < 0.00001 compared to the untreated controls.

The bone marrow of C57BL/6J mice aged between 6 and 8 weeks was flushed, followed by MSC isolation. As previously described [32,50,51], MSCs were seeded and assessed for cell viability and proliferation (Figure 6). MSCs demonstrated a marginal increase in cell viability at a dose of 50% L-CM (Figure 6A). While not as extensively as in our MC3T3-E1 results, MSCs demonstrated a significant decrease in cell proliferation with higher doses of L-CM (Figure 6B). Together with the previous results, our data suggest that L-CM treatment negatively regulates OB proliferation. 

### 3.6. L-CM Treatment Decreased Early Differentiation and Collagen Production of Bone-Marrow-Derived Osteoblasts

**Figure 7:**
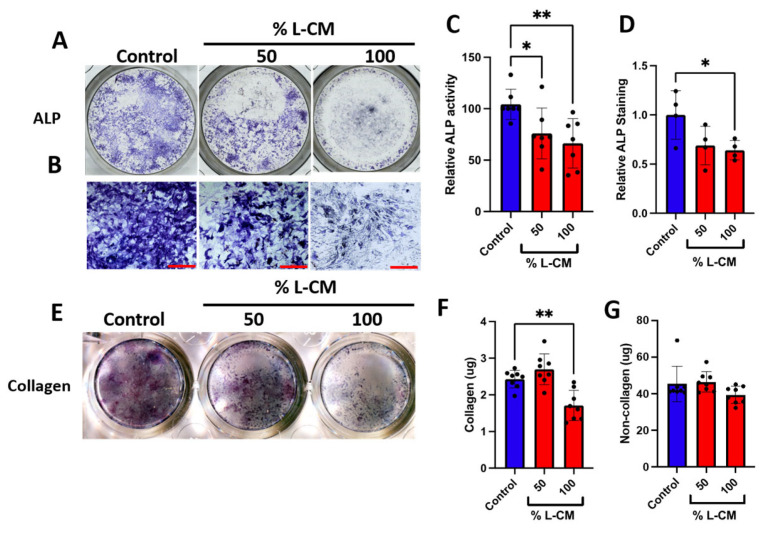
L-CM treatment decreased bone-marrow-derived osteoblast differentiation and function. Bone-marrow-derived MSCs were cultured with differentiation factors and treated with either 50 or 100% L-CM. After 1 week of culture, osteoblasts were stained for ALP (**A**,**B**). ALP activity (**C**) and staining were then quantified and assessed for each treatment (**D**). Collagen production was assessed through staining (**E**) and then quantified for the content of collagenous (**F**) and non-collagenous (**G**) proteins. N = 4–8. Data are presented as the mean ± SEM. * *p* < 0.05 and ** *p* < 0.01 compared to the untreated controls. Scale bar: 1000 μm.

Bone-marrow-derived MSCs were treated with osteoblastic differentiation factors as previously described [30,32,33] and cultured for one week. Next, we assessed early osteoblast differentiation and collagen production in cultures treated with 50 and 100% L-CM based on the findings above. While previous work showed no significant differences in differentiated osteoblasts after two days of culture [26], here, we assessed the effects of L-CM treatment after 7 days of differentiation, as previously described [30,33,43,52,53]. Similarly to the above findings, cultures were stained for ALP (Figure 7A,B). Our results showed a significant decrease in ALP activity and staining (Figure 7C,D). In addition, the quantification of collagen staining showed a decrease in collagenous protein production (Figure 7F). In contrast to our MC3T3-E1 results, the non-collagenous protein assessment did not reveal a significant difference between the L-CM-treated groups and the control (Figure 7G). Taken together, these results demonstrate that L-CM treatment led to decreased osteoblast differentiation and collagen production.

### 3.7. L-CM Treatment Decreased Bone-Marrow-Derived Osteoblast Matrix Mineral Deposition

**Figure 8:**
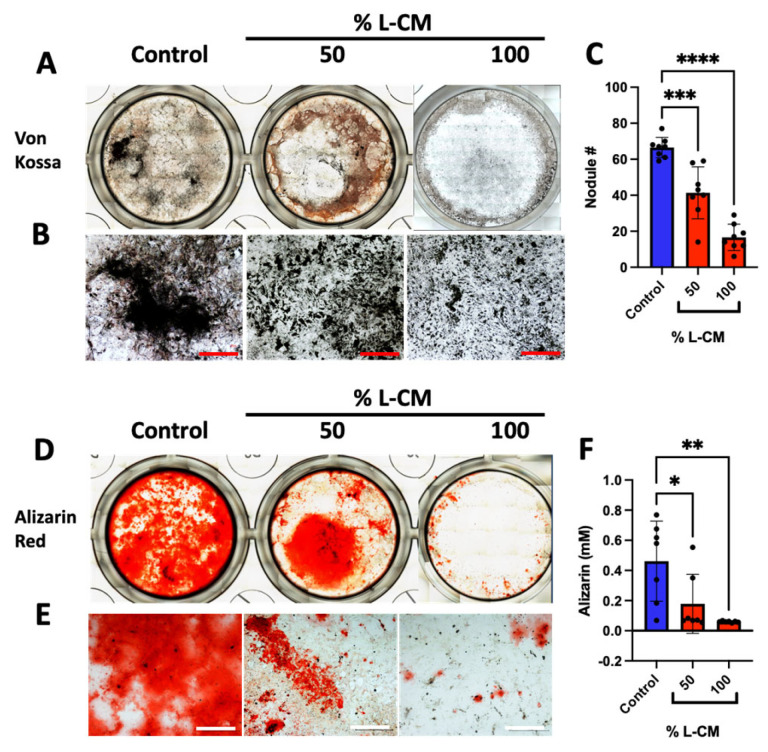
L-CM treatment decreased bone-marrow-derived osteoblast matrix mineral deposition. Bone-marrow-derived MSCs were cultured with differentiation factors and treated with either 50 or 100% L-CM. After 3 weeks in culture, osteoblasts were assessed for the nodule number and mineral deposition. A Von Kossa assay was used to stain deposits of calcium phosphate (**A**,**B**), followed by the quantification of nodule numbers (**C**). Calcium deposition was determined through Alizarin Red staining (**D**,**E**) and quantification (**F**). N = 3. Data are presented as the mean ± SEM. * *p* < 0.05, ** *p* < 0.01, *** *p* < 0.001, and **** *p* < 0.0001 compared to the untreated controls. Scale bar: 1000 μm.

After three weeks of culture, bone-marrow-derived osteoblasts were assessed for mineral deposition, as shown for the MC3T3-E1 cultures (Figure 4). Von Kossa staining was decreased with the L-CM treatment in a dose-dependent manner (Figure 8A,B). The quantification of nodule numbers was decreased in response to the L-CM treatment in a dose-dependent manner in comparison to the untreated controls. These results followed a similar trend to that of our MC3T3-E1 results (Figure 8C). In addition, Alizarin Red staining and quantification also showed a significant decrease in calcium deposition in the L-CM-treated culture in comparison to the untreated controls (Figure 8D–F). 

### 3.8. Co-Culture with Lymphatic Endothelial Cells Inhibited Osteoblast Differentiation

**Figure 9:**
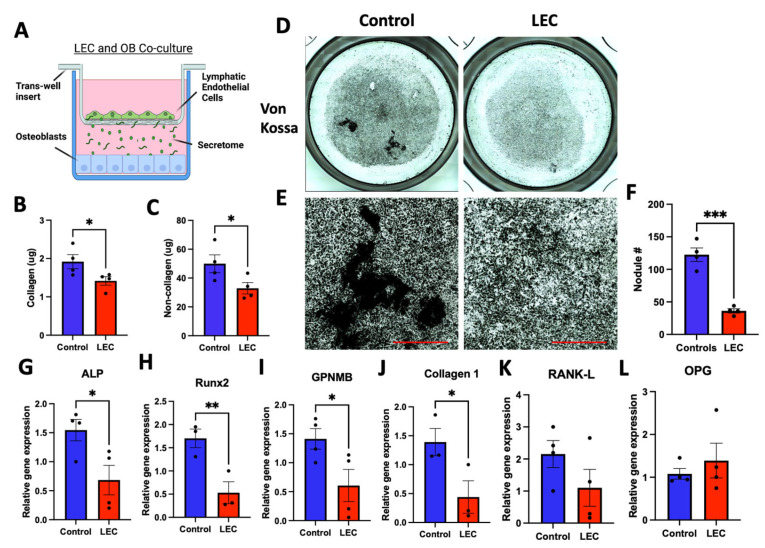
Co-culture of LECs with differentiating osteoblasts using a trans-well system led to decreased differentiation. MC3T3-E1 cells were cultured with differentiation factors and exposed to the lymphatic secretome from LECs seeded in a trans-well carrier (**A**). After 1 week of culture, osteoblast matrices were assessed for collagenous (**B**) and non-collagenous (**C**) content. After 3 weeks of culture, Von Kossa staining was used to highlight deposits of calcium and potassium (**D**,**E**), and this was followed by nodule quantification (**F**). The gene expression of osteoblast-related markers was assessed after three weeks of culture (**G**–**L**). N = 3–4. Data are presented as the mean ± SEM. * *p* < 0.05, ** *p* < 0.01, and *** *p* < 0.001 compared to the untreated control. Scale bar: 1000 μm.

To further support the detrimental effect that the lymphatic secretome had on osteoblast differentiation, we co-cultured lymphatic endothelial cells (LECs) and MC3T3-E1 cells using a trans-well system as previously described [54,55,56] (Figure 9A). After one week of differentiation, collagenous and non-collagenous proteins were both decreased in the LEC co-culture group in comparison to the control (Figure 9B,C). After 3 weeks of culture, mineral staining (Figure 9D,E) marked a significant decrease in mineral content and nodule formation (Figure 9F). Gene expression analysis of the osteoblast-related markers ALP, Runx2, GPNMB, and collagen 1 showed that they were significantly decreased in the co-culture group when compared to the control (Figure 9G–J). We did not observe a difference in RANK-L or OPG gene expression (Figure 9K,L).

### 3.9. L-CM Inhibited Bone Formation in Calvaria Organ Culture

**Figure 10:**
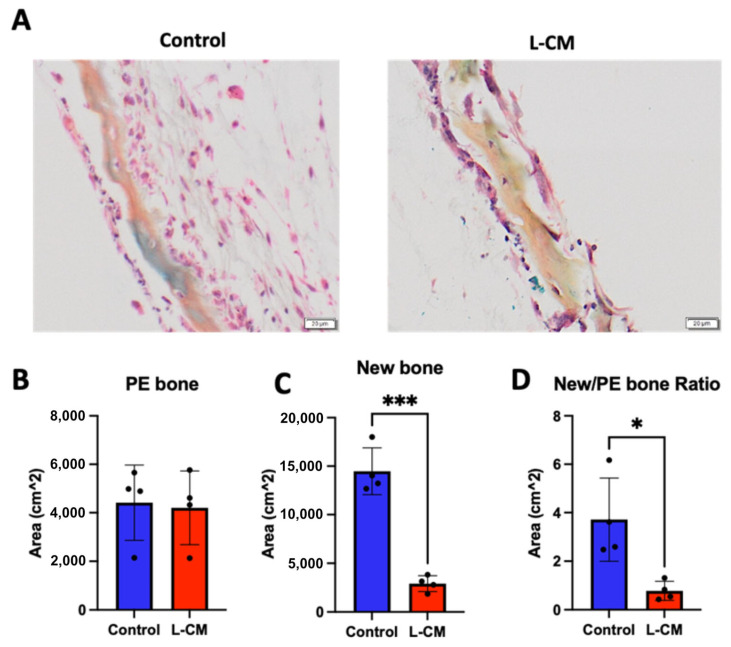
L-CM inhibited new bone formation in calvaria organ culture. Neonatal mouse calvariae were isolated, grown in culture, and treated with a control or 50% L-CM medium. After a week in culture, the calvariae were fixed and embedded for histological processing. Using modified hematoxylin and eosin staining, preexisting (PE) (orange) and newly formed bone (pink) was identified (**A**). The areas of PE (**B**) and new bone (**C**) were quantified, and the ratio of new/PE bone was presented (**D**). N = 4. Data are presented as the mean ± SEM. * *p* < 0.05 and *** *p* < 0.001 compared to the untreated controls. Scale bar: 20 μm.

The data presented above show that the lymphatic secretome was able to reduce OB proliferation and differentiation, but they are limited due to their in vitro nature. For this reason, we proceeded to determine the effect of L-CM treatment on bone formation using an explant culture of neonatal calvariae as previously described [37]. After one week of L-CM treatment, we were able to observe new bone formation in untreated calvariae compared to the L-CM-treated cultures (Figure 10A). The preexisting (PE) bone area remained unaffected in both the control and L-CM-treated groups (Figure 10B). In contrast, new bone formation in the L-CM-treated calvariae was significantly reduced when compared to that in the untreated controls (Figure 10C). These results were sustained even when comparing the new/PE bone ratio (Figure 10D). Collectively, these data suggest that the factors secreted from lymphatic endothelial cells have a detrimental effect on osteoblast differentiation and bone formation in both in vitro and ex vivo cultures.

## 4. Discussion

**Figure 11:**
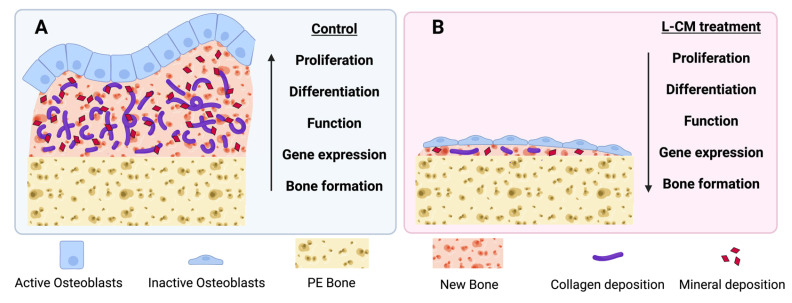
Schematic diagram on the effect of L-CM treatment on osteoblast differentiation and bone formation. Our results present a clear reduction in osteoblast proliferation, differentiation, function, and gene expression when treated with L-CM (**B**) in comparison to the untreated control (**A**). L-CM was also found to inhibit bone formation (**B**) when compared to the untreated control (**A**) in an ex vivo model for bone formation.

The goal of this study was to assess the effect that the lymphatic endothelial cell (LEC) secretome had on osteoblast differentiation and bone formation. Our results demonstrate that L-CM consistently inhibited osteoblast proliferation, differentiation, function, and gene expression. In addition, we found that L-CM directly inhibited bone formation in an explant culture (Figure 11). In contrast to previously reported studies [26], our data clearly showed that the direct influence of the LEC secretome led to a reduction in osteoblast differentiation and bone formation. Minimal to dramatic reduction in bone formation is one of the key features found in GSD osteopathies [2,3,4,5]. Although the mechanism of action was not explored in this study, we believe that our data provide the first line of evidence that the LEC secretome affects osteoblast proliferation, differentiation, and bone formation. These data will set the stage for future studies delineating the mechanism through which the LEC secretome reduces bone mass in CLAs.

The purpose of this study was to mimic the effect that lymphatics have on bone in CLAs. L-CM was collected from actively growing cultures that we predicted would mimic the expected proliferative profile of cells with PIK3CA or KRAS mutations. To date, the number of studies dissecting the osteopathic mechanism in CLAs is very limited. Until recent years, the bone phenotype in CLAs was poorly understood and was mainly limited to GSD patients who were diagnosed through their aggressive and localized bone loss [56,57,58]. Through histological staining of GSD bone samples, it is commonly reported that multiple osteoclasts are found at the site of bone resorption, unlike in unaffected bone [5]. In addition, pre-osteoclastic cells of GSD patients showed increased differentiation into mature and active osteoclasts in comparison with pre-osteoclastic cells derived from control subjects [28,59]. It is intriguing to assess the effects of the LEC secretome on osteoclast differentiation and bone resorption, an area that is under investigation by our group. Furthermore, microRNA analysis of GSD osteoclasts and osteoblasts revealed how some of these cells’ variations could be explained through changes in their miRNAome [59]. On the other hand, analysis of GSD-patient-derived osteoblasts revealed a decrease in differentiation and function [28]. Given the scarcity of data describing abnormalities in osteoblasts in CLAs, our studies provide unique insight into new information on the role that LECs play during lymphangiogenesis into bone.

While bone homeostasis in GSD patients appears to lead to bone resorption, there have been no mutations identified in patient bone cells to date. The only mutations described in CLAs are associated with their abnormal lymphatic endothelial cells [5,7,60,61]. These mutations, which are typically in genes involved in the RAS/MEK or PIK3CA/mTOR pathways [60,61,62], facilitate lymphatic invasion into bone. Direct engagement between lymphatics and bone through intratibial injection of normal LECs triggered massive bone loss in the injected bone [26]. Interestingly, lymphatic vessels were recently identified within normal bone and may play a role in CLA-related bone resorption [13]. Postnatally, lymphangiogenesis is largely absent unless prompted by exogenous stimuli, such as inflammation, cancer, and tissue repair [63,64,65]. For this reason, we collected L-CM from actively proliferating LECs to mimic the secretome of expanding lymphatics and assessed its effect on osteoblast differentiation and bone formation.

In order to study the effect of lymphatic endothelial cell conditioned medium (L-CM) on osteoblasts, we first delineated whether L-CM would induce cytotoxicity and/or reduce cell proliferation. We determined that L-CM is not cytotoxic to MC3T3-E1 or MSCs when quantifying the MTT signal (Figure 2A and Figure 6B). The MTT method was used to indirectly assess toxicity (mitochondrial activity) caused by the L-CM treatment. Since this was generally unchanged, we used the CyQuant assay to directly quantify the number of cells in the S phase and, therefore, provide a direct assessment of proliferation. Following this analysis, we identified that proliferation was inhibited in both MC3T3-E1 cells and MSCs in response to the L-CM treatment (Figure 2B and Figure 6B). The decrease in proliferation was more prominent in the MC3T3-E1 cell line than in the MSCs. This mild effect on MSC proliferation further highlights the role of L-CM in osteoblast differentiation (Figure 8 and Figure 9). A decrease in proliferation in these cells is known to directly inhibit cell differentiation, since it would limit cell growth into their respective nodule numbers [34]. Through these early results, we can already account for some of the inhibition in the osteoblastic response reported in GSD [28,59].

When treated with L-CM, early osteoblast differentiation was inhibited in a dose-dependent manner in MC3T3-E1 cells, primary cultures, and co-cultures (Figure 3, Figure 7 and Figure 9). MC3T3-E1 cells were more sensitive to L-CM inhibition, as reflected by the 80% drop in ALP staining and collagen matrix deposition at the highest treatment dose (Figure 3). It is of particular interest to note that non-collagenous proteins were also decreased in relation to collagenous proteins, which suggests an overall decrease in matrix deposition. When MSCs derived from GSD patients’ pathological bone were differentiated and assessed for ALP staining, these differentiated MSCs did not show a statistical difference when compared with those of control patients [28]. In our study, we observed a 40% drop in ALP staining and activity of bone-marrow-derived osteoblasts when treated with L-CM (Figure 7). In contrast to our MC3T3-E1 data, we observed an exclusive inhibition of collagenous proteins when treated with L-CM (Figure 7F,G). While our assay was sensitive to all collagenous proteins, osteoblasts are known to secrete vast amounts of type I collagen, as it is necessary for osteoblast maturation and function [40,66,67]. Even though non-collagenous proteins are still needed for proper osteoblast maturation, an inhibition of collagen type I secretion will lead to poor osteoblast differentiation [68,69]. When assessing late osteoblast differentiation associated with matrix mineralization and calcium deposition following treatment with L-CM, we observed a consistent decrease in mineral/calcium deposition in primary MC3T3-E1 and bone-marrow-derived osteoblast cultures (Figure 4 and Figure 8). Mineralized nodule formation and calcium deposition are commonly used to assess late osteoblast maturation in cell culture [39,70]. In both cultures, we found a decrease in the nodule number and calcium deposition of at least 70% when comparing the L-CM treatment with control cultures. These results further support previous findings that presented a marked decrease in the mineralization of GSD patients’ osteoblasts [28]. Together, these data highlight how the LEC secretome leads to the inhibition of MC3T3-E1 differentiation at higher doses of L-CM treatment and a likely delay in bone-marrow-derived osteoblast differentiation. This can be explained by the L-CM treatment having a more drastic effect on MSCs that have not yet been committed for osteoblast differentiation in comparison with actively differentiating osteoblasts. Further elucidation of this possible mechanism requires further investigation.

The gene expression of osteoblast-related markers was assessed during treatment with 25 and 50% L-CM (Figure 5), since these doses consistently decreased OB differentiation (Figure 2, Figure 3, Figure 7 and Figure 8). Treatment with L-CM caused a dose-dependent decrease in ALP and GPNMB mRNA expression during OB differentiation (Figure 5A–F). Inhibition of these genes is closely correlated with decreased OB differentiation, as previously described [33,38,45,51]. Interestingly, Runx2 and collagen 1 mRNA expression was only inhibited during early OB differentiation (week 1) when treated with 50% L-CM (Figure 5G,K). It is already known that the reduction in the expression of Runx2, an essential transcription factor for OB maturation, leads to the inhibition of OB differentiation [43]. Furthermore, the inhibition of collagen I expression directly hampers matrix production and leads to dysfunctional OB function [66,68,69]. Lastly, we found RANK-L to be tightly regulated by the L-CM treatment. The decreased RANK-L mRNA expression at week one in culture was correlated with the observed inhibition of ALP mRNA expression in response to the L-CM treatment (Figure 5A,M). This response was then reversed in weeks 2 and 3, suggesting an increase in RANK-L production in late OB differentiation (Figure 5N,M). Together, these data emphasize a marked decrease in OB maturation that would lead to a halt in bone formation. In addition, the increase in RANK-L production (osteoclast growth factor) in response to the L-CM treatment might explain the osteopathic phenotype observed in CLAs.

Previous work has shown that intratibial injection of LECs leads to aberrant bone loss [26]. In order to determine whether this phenomenon requires cell-to-cell interaction, we established a lymphatic-to-osteoblast co-culture model. Using a trans-well system, differentiating osteoblasts were actively influenced by the consistent secretion of the lymphatic secretome from growing LECs (Figure 9). Early differentiation of MC3T3-E1 cells in the co-culture system mimicked our data from the direct L-CM treatment in OBs (Figure 3 and Figure 7) through the inhibition of collagenous and non-collagenous proteins (Figure 9A,B). Similar results were obtained when assessing late OB differentiation (Figure 4, Figure 8 and Figure 9). Von Kossa staining demonstrated a marked decrease in mineral secretion (Figure 9C,D) with a decreased nodule number (Figure 9E) in the LEC-treated group in comparison with the control. In addition, the gene expression of osteoblast-related markers (ALP, Runx2, GPNMB, type 1 collagen) was also inhibited by the lymphatic co-culture (Figure 9G,J). Through this novel approach, we are the first group to demonstrate how the lymphatic secretome alone can significantly inhibit osteoblast differentiation in vitro.

To further support our studies, we assessed the effects of L-CM on a neonatal calvaria explant culture during active bone formation (Figure 10). Previous studies demonstrated how intratibial injection of normal LECs led to massive bone loss in mice [26]. Through our studies, we were able to determine that lymphatic-secreted factors alone were capable of greatly inhibiting new bone formation (Figure 10C). Interestingly, L-CM treatment alone did not alter preexisting bone (Figure 10B). This phenomenon is likely due to the lack of activated osteoclasts within the calvariae.

From our observations, we conclude that L-CM inhibits osteoblast differentiation and function, as assessed through MC3T3-E1 and bone-marrow-derived cultures. In addition, we also demonstrated how L-CM can prevent bone formation using a neonatal murine organ culture. Furthermore, the L-CM inhibition of OB differentiation could be caused by decreased OB proliferation. This was further confirmed by the sudden arrest of bone formation in the presence of lymphatic factors. Together, these results highlight the radical interaction between lymphatics and bone and how the lymphatic secretome alone may lead to a complete inhibition of new bone formation (Figure 11). While we have previously hypothesized that known secreted proteins are capable of triggering a signaling cascade when bone and lymphatics meet [1], we did not describe OB function during lymphatic invasion in bone. Our future studies are aimed at identifying the key factors within L-CM that are responsible for the bone cell dysregulation seen in CLAs.

## Figures and Tables

**Table 1:** RT-qPCR mouse (m) primers.

Gene Name	Sequence
m_18s_F	CTTAGAGGGACAAGTGGCG
m_18s_R	ACGCTGAGCCAGTCAGTGTA
m_ALP_F	CCAACTCTTTTGTGCCAGAGA
m_ALP_R	GGCTACATTGGTGTTGAGCTTTT
m_Col1 A1_F	GCTCCTCTTAGGGGCCACT
m_Col1 A1_R	ATTGGGGACCCTTAGGCCAT
m_GPNMB_F	AATGGGTCTGGCACCTACTG
m_GPNMB_R	GGCTTGTACGCCTTGTGTTT
m_OPG_R	AGCAGGAGTGCAACCGCACC
m_OPG_F	TTCCAGCTTGCACCACGCCG
m_RANK-L_F	GCTCCGAGCTGGTGAAGAAA
m_RANK-L_R	CCCCAAAGTACGTCGCATCT
m_Runx2_F	GACTGTGGTTACCGTCATGGC
m_Runx2_R	ACTTGGTTTTTCATAACAGCGGA

## Data Availability

Not applicable.

## References

[1] Solorzano E., Alejo A.L., Ball H.C., Magoline J., Khalil Y., Kelly M., Safadi F.F. (2022). Osteopathy in Complex Lymphatic Anomalies. Int. J. Mol. Sci..

[2] Heffez L., Doku H.C., Carter B.L., Feeney J.E. (1983). Perspectives on massive osteolysis. Report of a case and review of the literature. Oral. Surg. Oral. Med. Oral. Pathol..

[3] Saify F.Y., Gosavi S.R. (2014). Gorham’s disease: A diagnostic challenge. J. Oral. Maxillofac. Pathol..

[4] Nikolaou V.S., Chytas D., Korres D., Efstathopoulos N. (2014). Vanishing bone disease (Gorham-Stout syndrome): A review of a rare entity. World J. Orthop..

[5] Angelini A., Mosele N., Pagliarini E., Ruggieri P. (2022). Current concepts from diagnosis to management in Gorham-Stout disease: A systematic narrative review of about 350 cases. EFORT Open Rev..

[6] Homayun-Sepehr N., McCarter A.L., Helaers R., Galant C., Boon L.M., Brouillard P., Vikkula M., Dellinger M.T. (2021). KRAS-driven model of Gorham-Stout disease effectively treated with trametinib. JCI Insight.

[7] Manevitz-Mendelson E., Leichner G.S., Barel O., Davidi-Avrahami I., Ziv-Strasser L., Eyal E., Pessach I., Rimon U., Barzilai A., Hirshberg A. (2018). Somatic NRAS mutation in patient with generalized lymphatic anomaly. Angiogenesis.

[8] Kato H., Ozeki M., Fukao T., Matsuo M. (2017). MR imaging findings of vertebral involvement in Gorham-Stout disease, generalized lymphatic anomaly, and kaposiform lymphangiomatosis. Jpn. J. Radiol..

[9] Trenor C.C., Chaudry G. (2014). Complex lymphatic anomalies. Semin. Pediatr. Surg..

[10] Alderfer L., Wei A., Hanjaya-Putra D. (2018). Lymphatic Tissue Engineering and Regeneration. J. Biol. Eng..

[11] Azzali G. (1982). Transendothelial transport of lipids in the absorbing lymphatic vessel. Experientia.

[12] Oliver G., Harvey N. (2002). A stepwise model of the development of lymphatic vasculature. Ann. N. Y. Acad. Sci..

[13] Biswas L., Chen J., De Angelis J., Singh A., Owen-Woods C., Ding Z., Pujol J.M., Kumar N., Zeng F., Ramasamy S.K. (2023). Lymphatic vessels in bone support regeneration after injury. Cell.

[14] Afshari A., Shamdani S., Uzan G., Naserian S., Azarpira N. (2020). Different approaches for transformation of mesenchymal stem cells into hepatocyte-like cells. Stem Cell Res. Ther..

[15] Chitteti B.R., Cheng Y.H., Streicher D.A., Rodriguez-Rodriguez S., Carlesso N., Srour E.F., Kacena M.A. (2010). Osteoblast lineage cells expressing high levels of Runx2 enhance hematopoietic progenitor cell proliferation and function. J. Cell Biochem..

[16] Amarasekara D.S., Kim S., Rho J. (2021). Regulation of Osteoblast Differentiation by Cytokine Networks. Int. J. Mol. Sci..

[17] McDonald M.M., Khoo W.H., Ng P.Y., Xiao Y., Zamerli J., Thatcher P., Kyaw W., Pathmanandavel K., Grootveld A.K., Moran I. (2021). Osteoclasts recycle via osteomorphs during RANKL-stimulated bone resorption. Cell.

[18] Kim J.M., Lin C., Stavre Z., Greenblatt M.B., Shim J.H. (2020). Osteoblast-Osteoclast Communication and Bone Homeostasis. Cells.

[19] Hirayama T., Sabokbar A., Itonaga I., Watt-Smith S., Athanasou N.A. (2001). Cellular and humoral mechanisms of osteoclast formation and bone resorption in Gorham–Stout disease. J. Pathol..

[20] Möller G., Priemel M., Amling M., Werner M., Kuhlmey A.S., Delling G. (1999). The Gorham-Stout syndrome (Gorham’s massive osteolysis). J. Bone Jt. Surg. Br. Vol..

[21] Edwards J.R., Williams K., Kindblom L.G., Meis-Kindblom J.M., Hogendoorn P.C., Hughes D., Forsyth R.G., Jackson D., Athanasou N.A. (2008). Lymphatics and bone. Hum. Pathol..

[22] Zhu Y., Wu Y., Liang Y., Tan W., Liu Z., Xiao J. (2016). Regulation of expression level of fms-like tyrosine kinase-4 is related to osteoclast differentiation. Arch. Med. Sci..

[23] Jones D., Min W. (2011). An overview of lymphatic vessels and their emerging role in cardiovascular disease. J. Cardiovasc. Dis. Res..

[24] Monroy M., McCarter A.L., Hominick D., Cassidy N., Dellinger M.T. (2020). Lymphatics in bone arise from pre-existing lymphatics. Development.

[25] Edwards J., Schulze E., Sabokbar A., Gordon-Andrews H., Jackson D., Athanasou N.A. (2008). Absence of lymphatics at the bone-implant interface: Implications for periprosthetic osteolysis. Acta Orthop..

[26] Wang W., Wang H., Zhou X., Li X., Sun W., Dellinger M., Boyce B.F., Xing L. (2019). Lymphatic Endothelial Cells Produce M-CSF, Causing Massive Bone Loss in Mice. J. Bone Miner. Res. Off. J. Am. Soc. Bone Miner. Res..

[27] Devlin R.D., Bone H.G., Roodman G.D. (1996). Interleukin-6: A potential mediator of the massive osteolysis in patients with Gorham-Stout disease. J. Clin. Endocrinol. Metab..

[28] Rossi M., Buonuomo P.S., Battafarano G., Conforti A., Mariani E., Algeri M., Pelle S., D’Agostini M., Macchiaiolo M., De Vito R. (2020). Dissecting the mechanisms of bone loss in Gorham-Stout disease. Bone.

[29] Brodszki N., Länsberg J.K., Dictor M., Gyllstedt E., Ewers S.B., Larsson M.K., Eklund E.A. (2011). A novel treatment approach for paediatric Gorham-Stout syndrome with chylothorax. Acta Paediatr..

[30] Abdelmagid S.M., Belcher J.Y., Moussa F.M., Lababidi S.L., Sondag G.R., Novak K.M., Sanyurah A.S., Frara N.A., Razmpour R., Del Carpio-Cano F.E. (2014). Mutation in osteoactivin decreases bone formation in vivo and osteoblast differentiation in vitro. Am. J. Pathol..

[31] Ball H.C., Moussa F.M., Mbimba T., Orman R., Safadi F.F., Cooper L.N. (2016). Methods and insights from the characterization of osteoprogenitor cells of bats (Mammalia: Chiroptera). Stem Cell Res..

[32] Moussa F.M., Cook B.P., Sondag G.R., DeSanto M., Obri M.S., McDermott S.E., Safadi F.F. (2021). The role of miR-150 regulates bone cell differentiation and function. Bone.

[33] Abdelmagid S.M., Barbe M.F., Rico M.C., Salihoglu S., Arango-Hisijara I., Selim A.H., Anderson M.G., Owen T.A., Popoff S.N., Safadi F.F. (2008). Osteoactivin, an anabolic factor that regulates osteoblast differentiation and function. Exp. Cell Res..

[34] Safadi F.F., Xu J., Smock S.L., Kanaan R.A., Selim A.H., Odgren P.R., Marks S.C., Owen T.A., Popoff S.N. (2003). Expression of connective tissue growth factor in bone: Its role in osteoblast proliferation and differentiation in vitro and bone formation in vivo. J. Cell. Physiol..

[35] Sondag G.R., Mbimba T.S., Moussa F.M., Novak K., Yu B., Jaber F.A., Abdelmagid S.M., Geldenhuys W.J., Safadi F.F. (2016). Osteoactivin inhibition of osteoclastogenesis is mediated through CD44-ERK signaling. Exp. Mol. Med..

[36] Liu B., Lu Y., Wang Y., Ge L., Zhai N., Han J. (2019). A protocol for isolation and identification and comparative characterization of primary osteoblasts from mouse and rat calvaria. Cell Tissue Bank..

[37] Mohammad K.S., Chirgwin J.M., Guise T.A., Westendorf J.J. (2008). Assessing New Bone Formation in Neonatal Calvarial Organ Cultures. Osteoporosis: Methods and Protocols.

[38] Alborzi A., Mac K., Glackin C.A., Murray S.S., Zernik J.H. (1996). Endochondral and intramembranous fetal bone development: Osteoblastic cell proliferation, and expression of alkaline phosphatase, m-twist, and histone H4. J. Craniofacial Genet. Dev. Biol..

[39] Morais S., Carvalho G.S., Faria J.L., Gomes H.T., Sousa J.P. (1998). In vitro biomineralization by osteoblast-like cells. II. Characterization of cellular culture supernatants. Biomaterials.

[40] Lynch M.P., Stein J.L., Stein G.S., Lian J.B. (1995). The influence of type I collagen on the development and maintenance of the osteoblast phenotype in primary and passaged rat calvarial osteoblasts: Modification of expression of genes supporting cell growth, adhesion, and extracellular matrix mineralization. Exp. Cell Res..

[41] Bills C.E., Eisenberg H., Pallante S.L. (1974). Complexes of organic acids with calcium phosphate: The Von Kossa stain as a clue to the composition of bone mineral. Johns Hopkins Med. J..

[42] Driessens F.C., van Dijk J.W., Borggreven J.M. (1978). Biological calcium phosphates and their role in the physiology of bone and dental tissues I. Composition and solubility of calcium phosphates. Calcif. Tissue Res..

[43] Prince M., Banerjee C., Javed A., Green J., Lian J.B., Stein G.S., Bodine P.V., Komm B.S. (2001). Expression and regulation of Runx2/Cbfa1 and osteoblast phenotypic markers during the growth and differentiation of human osteoblasts. J. Cell Biochem..

[44] Bateman J.P., Safadi F.F., Susin C., Wikesjo U.M. (2012). Exploratory study on the effect of osteoactivin on bone formation in the rat critical-size calvarial defect model. J. Periodontal Res..

[45] Frara N., Abdelmagid S.M., Sondag G.R., Moussa F.M., Yingling V.R., Owen T.A., Popoff S.N., Barbe M.F., Safadi F.F. (2016). Transgenic Expression of Osteoactivin/gpnmb Enhances Bone Formation In Vivo and Osteoprogenitor Differentiation Ex Vivo. J. Cell. Physiol..

[46] Abdelmagid S.M., Sondag G.R., Moussa F.M., Belcher J.Y., Yu B., Stinnett H., Novak K., Mbimba T., Khol M., Hankenson K.D. (2015). Mutation in Osteoactivin Promotes Receptor Activator of NFkappaB Ligand (RANKL)-mediated Osteoclast Differentiation and Survival but Inhibits Osteoclast Function. J. Biol. Chem..

[47] Takahashi N., Udagawa N., Suda T. (1999). A new member of tumor necrosis factor ligand family, ODF/OPGL/TRANCE/RANKL, regulates osteoclast differentiation and function. Biochem. Biophys. Res. Commun..

[48] Gori F., Hofbauer L.C., Dunstan C.R., Spelsberg T.C., Khosla S., Riggs B.L. (2000). The expression of osteoprotegerin and RANK ligand and the support of osteoclast formation by stromal-osteoblast lineage cells is developmentally regulated. Endocrinology.

[49] Schneider K.N., Masthoff M., Gosheger G., Klingebiel S., Schorn D., Roder J., Vogler T., Wildgruber M., Andreou D. (2020). Gorham-Stout disease: Good results of bisphosphonate treatment in 6 of 7 patients. Acta Orthop..

[50] Arosarena O.A., Barr E.W., Thorpe R., Yankey H., Tarr J.T., Safadi F.F. (2018). Osteoactivin regulates head and neck squamous cell carcinoma invasion by modulating matrix metalloproteases. J. Cell. Physiol..

[51] Yu B., Sondag G.R., Malcuit C., Kim M.H., Safadi F.F. (2016). Macrophage-Associated Osteoactivin/GPNMB Mediates Mesenchymal Stem Cell Survival, Proliferation, and Migration Via a CD44-Dependent Mechanism. J. Cell Biochem..

[52] Blair H.C., Larrouture Q.C., Li Y., Lin H., Beer-Stoltz D., Liu L., Tuan R.S., Robinson L.J., Schlesinger P.H., Nelson D.J. (2017). Osteoblast Differentiation and Bone Matrix Formation In Vivo and In Vitro. Tissue Eng. Part. B Rev..

[53] Karner C.M., Long F.X. (2017). Wnt signaling and cellular metabolism in osteoblasts. Cell Mol. Life Sci..

[54] Lee J., Park S., Roh S. (2015). Transdifferentiation of mouse adipose-derived stromal cells into acinar cells of the submandibular gland using a co-culture system. Exp. Cell Res..

[55] Nicolin V., Bortul R., Bareggi R., Baldini G., Martinelli B., Narducci P. (2008). Breast adenocarcinoma MCF-7 cell line induces spontaneous osteoclastogenesis via a RANK-ligand-dependent pathway. Acta Histochem..

[56] Kumar R., Harris-Hooker S., Kumar R., Sanford G. (2011). Co-culture of Retinal and Endothelial Cells Results in the Modulation of Genes Critical to Retinal Neovascularization. Vasc. Cell.

[57] Gem M., Ozkul E., Arslan H. (2014). Gorham-Stout’s disease in the metatarsus: A case report. Acta Orthop. Traumatol. Turc..

[58] Tena-Sanabria M.E., Jesús-Mejenes L.Y., Fuentes-Herrera G., Álvarez-Martínez F.A., Victorio-García N.P., Núñez-Enríquez J.C. (2019). A report of two children with Gorham-Stout disease. BMC Pediatr..

[59] Rossi M., Rana I., Buonuomo P.S., Battafarano G., Mariani E., D’Agostini M., Porzio O., De Martino V., Minisola S., Macchiaiolo M. (2021). Dysregulated miRNAs in bone cells of patients with Gorham-Stout disease. FASEB J..

[60] Li D., March M.E., Gutierrez-Uzquiza A., Kao C., Seiler C., Pinto E., Matsuoka L.S., Battig M.R., Bhoj E.J., Wenger T.L. (2019). ARAF recurrent mutation causes central conducting lymphatic anomaly treatable with a MEK inhibitor. Nat. Med..

[61] Rodriguez-Laguna L., Agra N., Ibanez K., Oliva-Molina G., Gordo G., Khurana N., Hominick D., Beato M., Colmenero I., Herranz G. (2019). Somatic activating mutations in PIK3CA cause generalized lymphatic anomaly. J. Exp. Med..

[62] Ozeki M., Aoki Y., Nozawa A., Yasue S., Endo S., Hori Y., Matsuoka K., Niihori T., Funayama R., Shirota M. (2019). Detection of NRAS mutation in cell-free DNA biological fluids from patients with kaposiform lymphangiomatosis. Orphanet J. Rare Dis..

[63] Cueni L.N., Detmar M. (2006). New insights into the molecular control of the lymphatic vascular system and its role in disease. J. Investig. Dermatol..

[64] Wang Y., Oliver G. (2010). Current views on the function of the lymphatic vasculature in health and disease. Genes. Dev..

[65] Christiansen A., Detmar M. (2011). Lymphangiogenesis and cancer. Genes. Cancer.

[66] Shi S., Kirk M., Kahn A.J. (1996). The role of type I collagen in the regulation of the osteoblast phenotype. J. Bone Miner. Res. Off. J. Am. Soc. Bone Miner. Res..

[67] Aubin J.E., Liu F., Malaval L., Gupta A.K. (1995). Osteoblast and chondroblast differentiation. Bone.

[68] Mizuno M., Fujisawa R., Kuboki Y. (2000). Type I collagen-induced osteoblastic differentiation of bone-marrow cells mediated by collagen-alpha2beta1 integrin interaction. J. Cell. Physiol..

[69] Maehata Y., Lee M.-C.-i., Hata R.-I. (2009). Roles of Collagen Molecules in Growth and Differentiation of Human Osteoblasts. J. Oral. Biosci..

[70] Patntirapong S., Chanruangvanit C., Lavanrattanakul K., Satravaha Y. (2021). Assessment of bisphosphonate treated-osteoblast behaviors by conventional assays and a simple digital image analysis. Acta Histochem..

